# Controlling stable Bloch points with electric currents

**DOI:** 10.1038/s41598-023-45111-5

**Published:** 2023-11-02

**Authors:** Martin Lang, Swapneel Amit Pathak, Samuel J. R. Holt, Marijan Beg, Hans Fangohr

**Affiliations:** 1 Faculty of Engineering and Physical Sciences, University of Southampton, Southampton, SO17 1BJ UK; 2 Max Planck Institute for the Structure and Dynamics of Matter, Luruper Chaussee 149, 22761 Hamburg, Germany; 3 Department of Earth Science and Engineering, Imperial College London, London, SW7 2AZ UK; 4 Center for Free-Electron Laser Science, Luruper Chaussee 149, 22761 Hamburg, Germany

**Keywords:** Magnetic properties and materials, Magnetic properties and materials, Computational science

## Abstract

The Bloch point is a point singularity in the magnetisation configuration, where the magnetisation vanishes. It can exist as an equilibrium configuration and plays an important role in many magnetisation reversal processes. In the present work, we focus on manipulating Bloch points in a system that can host stable Bloch points—a two-layer FeGe nanostrip with opposite chirality of the two layers. We drive Bloch points using spin-transfer torques and find that Bloch points can move collectively without any Hall effect and report that Bloch points are repelled from the sample boundaries and each other. We study pinning of Bloch points at wedge-shaped constrictions (notches) in the nanostrip and demonstrate that arrays of Bloch points can be moved past a series of notches in a controlled manner by applying consecutive current pulses of different strength. Finally, we simulate a T-shaped geometry and demonstrate that a Bloch point can be moved along different paths by applying current between suitable strip ends.

Bloch points^1,2^ are point singularities that can be observed in magnetic systems. They have first been studied in the context of bubble memories^3^. Bloch points play an important role in many dynamical processes, such as vortex–antivortex annihilation^4^, vortex core reversal^5^, and skyrmion reversal^6^. Recently, their static microscopic 3D structure^7–9^ and their dynamics^10,11^ were measured experimentally.

Beg et al.^12^ demonstrated that a stable Bloch point can exist in a thin helimagnetic cylinder consisting of two layers with opposite chirality. The Bloch point in this system is of circulating type and exists in two different configurations, head-to-head (HH) and tail-to-tail (TT). In a recent work^13^, we have demonstrated that rectangular two-layer nanostrips with opposite chirality can host multiple Bloch points, and in a nanostrip of suitable size any combination of Bloch points of the two types can be in equilibrium, which is necessary for encoding data with Bloch points.

The magnetisation around a Bloch point spans the entire solid angle. In the chiral two-layer system we observe circulating Bloch points. The circulation of the in-plane magnetisation is coupled to the polarisation of the two vortices enclosing a Bloch point via the material chirality. Because of the opposite chirality in the two layers we obtain vortices with opposite polarisation and the same circularity (due to the exchange coupling). Examples of the HH Bloch point and the TT Bloch point in this system are shown in Fig. 1.

**Figure 1:**
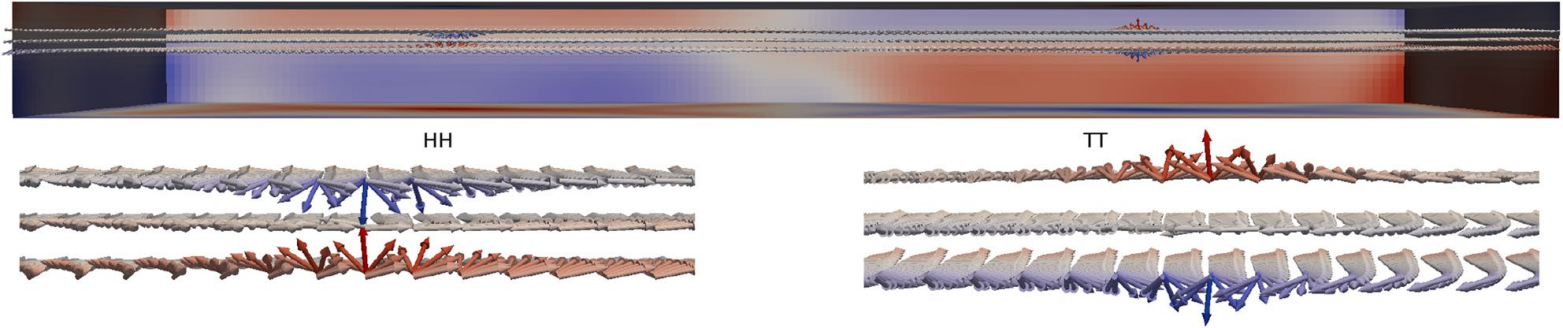
Magnetisation of a HH Bloch point (left half) and a TT Bloch point (right half) in a configuration containing two Bloch points. In the visualisation we have interpolated the magnetisation in the discretisation cells onto the discretisation cell nodes. Therefore, the middle layer of arrows is located directly at the interface of the two material layers at $z=0\,{\textrm{nm}}$.

In this work, we study the motion of one or multiple Bloch points under applied spin-transfer torques in a system consisting of two layers with opposite chirality. We find that the Bloch point moves without any Hall effect in the two-layer geometry. Multiple Bloch points move collectively, independent of their type or arrangement. We study the effect of geometry variations of the nanostrip by removing the magnetic material in one or multiple notches at the edge of the nanostrip. Finally, we simulate the motion of a Bloch point in a T-shaped geometry and show that we can control the motion of the Bloch point with the applied current. Depending on the current direction, the Bloch point can move along different paths, i.e. between arbitrary ends of the structure.

## Results

### Rectangular strips

**Figure 2:**
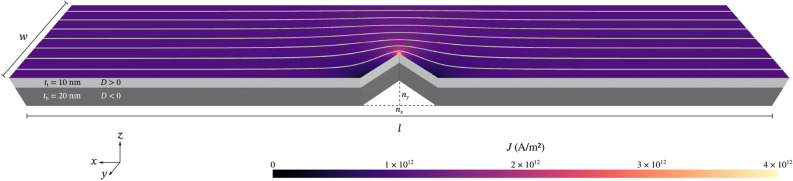
Geometry of the nanostrips studied in this work. All nanostrips consist of two layers with opposite chirality (light and dark grey), the interface is located at $z=0\,{\textrm{nm}}$. Length *l* and width *w* are adjusted as required. In a later part of this work we also study the effect of notches with width $n_x$ and depth $n_y$ in the strip. They extend throughout the whole sample thickness, as shown here. The colour and the streamlines on the top surface show the current density and direction in the nanostrip with a notch.

First, we focus on driving Bloch points in rectangular strips. We simulate nanostrips with length $l=1500\,{\textrm{nm}}$ or $l=2000\,{\textrm{nm}}$ and width $w=100\,{\textrm{nm}}$. The nanostrips consist of two layers with opposite material chirality, i.e. opposite sign of the Dzyaloshinkii–Moriya constant. The bottom layer has thickness $t_{\textrm{b}}=20\,{\textrm{nm}}$, the top layer thickness $t_{\textrm{t}}=10\,{\textrm{nm}}$, the interface is located at $z=0\,{\textrm{nm}}$. We show the geometry in Fig. 2, the two layers are indicated with dark and light grey. The figure shows a nanostrip with an additional notch studied later in this work. The top surface shows the corresponding simulated current density.

**Figure 3:**
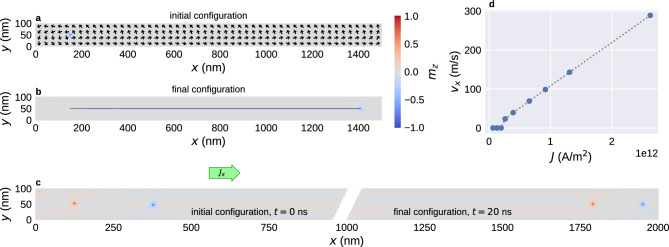
Movement of Bloch points with applied spin current through uniform nanostrips. (**a**,**b**) Movement of a single Bloch point with current density $J=0.7\times 10^{12}\,{\text {A/m}}^{2}$. The Bloch point is initialised near the left sample edge (**a**). When applying current, the Bloch point moves in $+x$ direction until it reaches the right sample edge (**b**). (**c**) Movement of two Bloch points of opposite type. (**d**) Speed of a single Bloch point depending on the current strength. A linear speed increase can be observed above a certain depinning threshold.

We begin by initialising the system and relaxing it to a state that contains one or two Bloch points near the left sample boundary and apply current in $+x$ direction. Figure 3a shows an initial configuration with a single Bloch point located at $x\approx 150\,{\textrm{nm}}$ in a $1500\,{\textrm{nm}}$ strip. The cross-section shows the magnetisation of the top material layer just above the interface of the two material layers (i.e. in the first layer of discretisation cells above the interface, at $z=1.25\,{\textrm{nm}}$), colour indicates the out-of-plane component $m_z$, and arrows the in-plane components $m_x$ and $m_y$. When current is applied in $+x$ direction, the Bloch point moves along the current direction until it reaches the right sample boundary, where it stops presumably due to the repulsion from the sample edge. Figure 3b shows the final configuration with the Bloch point at $x\approx 1400\,{\textrm{nm}}$. The blue line shows the path of the Bloch point. We can see that the Bloch point moves in a straight line and is not subject to any Hall effect, independent of the strength of the applied current. This is qualitatively different from the Bloch point in a chiral bobber studied in Ref.^14^, where the whole object is subject to a current-dependent Hall effect. We have also performed simulations for much wider strips ($w=600\,{\textrm{nm}}$) to verify that the straight motion is not caused by the repulsion from the sample boundaries in *y* direction.

Figure 3c shows an example for two Bloch points in the configuration tail-to-tail and head-to-head in a $2000\,{\textrm{nm}}$ strip. The left part shows the initial configuration with $x_{{\textrm{TT,i}}}\approx 125\,{\textrm{nm}}$ and $x_{{\textrm{HH,i}}}\approx 375\,{\textrm{nm}}$. The right part shows the final configuration with $x_{{\textrm{TT,f}}}\approx 1800\,{\textrm{nm}}$ and $x_{{\textrm{HH,f}}}\approx 1950\,{\textrm{nm}}$. Both Bloch points move along the current direction in a straight line. The Bloch-point type does not affect the motion. We obtain qualitatively similar results for configurations containing two Bloch points of the same type and for configurations containing more than two Bloch points.

Figure 3d shows the average speed of a single Bloch point as a function of the applied current density. We can observe pinning for small current densities and linearly increasing velocity for larger current densities. We cannot estimate the exact depinning current because it depends on the discretisation. Similar pinning behaviour of the Bloch point has been observed in other micromagnetic^5,10,14^ and atomistic^15,16^ simulations before. In the remainder of this work we focus on larger current densities above the depinning threshold.

### Nanostrip with one notch

**Figure 4:**
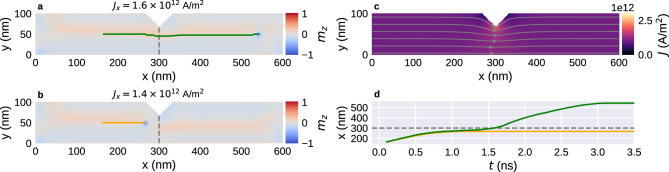
Motion of a Bloch point in a nanostrip ($l=600\,{\textrm{nm}}$, $w=100\,{\textrm{nm}}$) with a single notch under applied current. (**a**,**b**) Final configuration and Bloch-point trajectory for two different current strengths: (**a**) the Bloch point can move past the notch for $J_x=1.6\times 10^{12}\,{\text {A/m}}{}^2$; (**b**) the Bloch point is trapped at the notch for $J_x=1.4\times 10^{12}\,{\text {A/m}}{}^2$. (**c**) Simulated current profile. (**d**) Position of the Bloch points in (**a**) and (**b**) as a function of simulation time.

Next, we study the motion of a single Bloch point in a nanostrip with a notch. We simulate a strip with $l=600\,{\textrm{nm}}$ with a wedge-shaped notch at $x=300\,{\textrm{nm}}$, extending through the sample in *z* direction (Fig. 2). The notch tip is located at $y=70\,{\textrm{nm}}$ ($n_y=30\,{\textrm{nm}}$) and the opening angle is 90$^{\circ }$ ($n_x=60\,{\textrm{nm}}$). An *xy* cross-section is shown in Fig. 4. We use a finite-element simulation to compute the non-uniform current density profile in this geometry, more details are provided in the Methods section. The resulting current profile is shown in Fig. 4c. Near the notch we can observe a variation in the current density with the maximum at the tip of the notch.

The simulated current densities on the order of $10^{12}\,{\text {A/m}}^{2}$ would lead to a significant temperature increase due to Joule heating. A detailed study^17^ shows that the material, pulse duration, and cooling from the substrate play important roles in the control of the temperature. In this prototype study we ignore all temperature-related effects and possible engineering efforts that would need to be addressed for higher technical readiness levels.

First, we focus on two current densities $J_{x,\textrm{a}}=1.6\times 10^{12}\,{\text {A/m}}{}^2$ and $J_{x,\textrm{b}}=1.4\times 10^{12}\,{\text {A/m}}{}^2$. We initialise the systems with a Bloch point at $x_{\textrm{i}}\approx 165\,{\textrm{nm}}$. The final configurations with applied current are shown in Fig. 4a and b for $J_{x,\textrm{a}}$ and $J_{x,\textrm{b}}$, respectively. The solid lines show the Bloch-point path from its initial to its final configuration. Figure 4d shows the *x* position of the Bloch point in the two simulations as a function of simulation time. We observe repulsion of the Bloch point from the notch similar to the repulsion from the sample edge.

The current density $J_{x,\textrm{b}}$ (Fig. 4b) is not strong enough to overcome the repulsion from the notch and push the Bloch point past the notch. Instead, the Bloch point gets stuck near the notch at a final position $x_{{\textrm{f,b}}}\approx 270\,{\textrm{nm}}$, to which it moves in a straight line without any deflection in *y* direction. As a function of time (Fig. 4d), we can see a slow-down as the Bloch point approaches the notch. The motion stops around $t=1\,{\textrm{ns}}$: the applied current cannot push the Bloch point further against the restoring force from the notch constriction.

The current density $J_{x,\textrm{a}}$ (Fig. 4a) is strong enough to overcome the notch repulsion and push the Bloch point past the notch, and the Bloch point stops at $x_{{\textrm{f,a}}}\approx 550\,{\textrm{nm}}$ at around $t=3\,{\textrm{ns}}$ due to the edge repulsion of the sample at $x=600\,{\textrm{nm}}$. Near the notch, we can see a small displacement in the $-y$ direction, away from the tip of the notch. In the time-resolved data (Fig. 4d), we can see a slow-down of the Bloch point in front of the notch, very similar to the results for $J_{x,\textrm{a}}$ up to $t\approx 1\,{\textrm{ns}}$. The small deviations of the two curves for Bloch-point positions before the notch are caused by the slightly different current strengths. However, for the stronger current, the Bloch point continues to move towards the notch for $t>1\,{\textrm{ns}}$. We can see a slight speed increase around $t=1.3\,{\textrm{ns}}$, and the Bloch point passes the tip of the notch at around $t=1.6\,{\textrm{ns}}$ (and $x=300\,{\textrm{nm}}$ shown as a dashed line in Fig. 4). After passing the notch, we can see a strong speed increase and a fast motion in $+x$ direction until the Bloch point approaches the right sample boundary, where it slows down and eventually stops.

**Figure 5:**
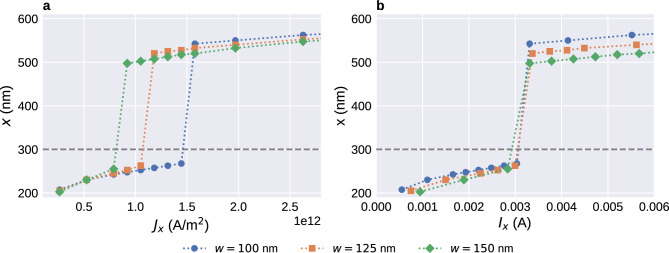
Final equilibrium position of a Bloch point in a strip with a notch as a function of applied current for three different strip widths. (**a**) The minimum required current density to move the Bloch point past the notch at $x=300\,{\textrm{nm}}$ increases with decreasing strip width. (**b**) The Bloch point moves past the notch at a critical total current that is independent of the details of the geometry. In both subfigures, we can see that narrower strips compress the overall structure and allow the Bloch point to move closer to the notch or sample edge.

To better understand the effect of the notch size on the pinning, we simulate strips with three different widths $w=100\,{\textrm{nm}}$, $w=125\,{\textrm{nm}}$, and $w=150\,{\textrm{nm}}$ for several different current densities. We keep the notch size of $n_y=30\,{\textrm{nm}}$ used before. Results are shown in Fig. 5.

Figure 5a shows the final position of the Bloch point as a function of current density. Final positions below the notch tip at $x=300\,{\textrm{nm}}$, visualised by the grey dashed line, mean that the Bloch point cannot move past the notch. Larger final positions mean that the Bloch point moves past the notch. We can see that the minimum current density required to move the Bloch point past the notch increases with decreasing strip width, as can be expected because the fraction by which the overall structure with the embedded Bloch point has to be compressed increases with decreasing strip width.

For large current densities, $J_x\ge 1.6\times 10^{12}\,{\text {A/m}}{}^2$, the Bloch point moves past the notch for all strip widths. Here, we can see that the Bloch point can move closer to the right sample boundary when the strip width decreases. This is presumably a result of the fact that the large-scale magnetisation configuration around the Bloch point is more compressed in narrower strips. It is in agreement with our previous work^13^, where we find that the optimal distance between Bloch points in a configuration containing multiple Bloch points also decreases with decreasing strip width. It appears that the large-scale configuration around the Bloch point wants to retain its approximately circular shape and reduces its radius due to the narrowness (in *y* direction) of the strip.

Figure 5b shows the final position as a function of total current through the *yz* plane at the notch tip, the minimum of the constriction. For similar geometries studied here, i.e. always a notch of the same size at the same *x* position, we find that the total current required to move the Bloch point past the notch is approximately independent of the strip width.

### Nanostrip with multiple notches

Based on the previous results, we can develop a protocol to move one or multiple Bloch points past a series of notches in a controlled manner. The overall idea is as follows. For weak current strengths, the Bloch point cannot move past a notch. Hence, we can use a weak current to move a Bloch point to a defined position close to a notch. Subsequently, we can apply a short, strong current pulse that pushes the Bloch point past the notch. Afterwards, we can let the system relax (i.e. switch off the current) or use a weak current to move the Bloch point to the next notch.

**Figure 6:**
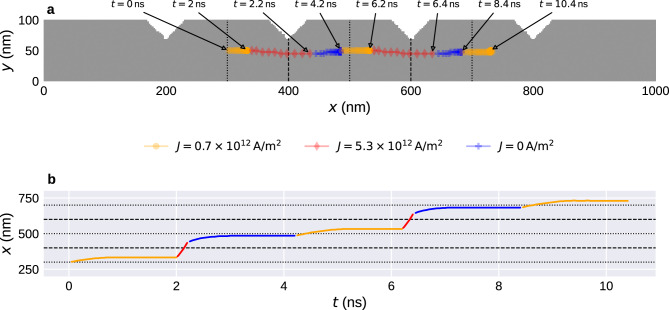
Driving a single Bloch point past several notches using a series of current pulses of different strength. (**a**) Strip geometry and trajectory of the Bloch point. (**b**) Position of the Bloch point in *x* direction as a function of simulation time. Dashed and dotted lines indicate the locations of the notches and centres of the wide areas in between, respectively.

Figure 6 demonstrates this process for a single Bloch point in a strip with length $l=1000\,{\textrm{nm}}$ and four notches at $x=200\,{\textrm{nm}}$, $x=400\,{\textrm{nm}}$, $x=600\,{\textrm{nm}}$, and $x=800\,{\textrm{nm}}$. We initialise the system in a configuration containing a single Bloch point between the first two notches at $x=300\,{\textrm{nm}}$. Figure 6a shows the strip geometry and the path of the Bloch point, Fig. 6b shows the *x* position of the Bloch point as a function of simulation time *t*.

We use a three-step process to move the Bloch point past a notch. (i) We apply a current $J_{x,(\textrm{i})}=0.7\times 10^{12}\,{\text {A/m}}{}^2$ to move the Bloch point to the next notch, where it gets trapped. We use a pulse length $\Delta t_{(\textrm{i})}=2\,{\textrm{ns}}$ for this alignment of the Bloch point to the left of the notch. From the time-resolved data we can see that the Bloch point stops moving after $\Delta t\approx 0.5\,{\textrm{ns}}$. The longer pulse duration can be useful to ensure that the Bloch point reaches the notch independent of its initial position. Applying the pulse for “too long” does not affect the configuration due to the trapping at the notch. (ii) We apply a strong pulse with $J_{x,({\textrm{ii}})}=5.3\times 10^{12}\,{\text {A/m}}{}^2$ with pulse length $\Delta t_{({\textrm{ii}})}=0.2\,{\textrm{ns}}$. This pulse pushes the Bloch point past the notch. A short pulse duration is required to ensure that the Bloch point only moves past one notch. (iii) We remove the current and let the system relax for $\Delta t_{({\textrm{iii}})}=2\,{\textrm{ns}}$. During this period, the Bloch point moves away from the notch until it reaches its equilibrium position near the centre of the region between the two notches, hereinafter called storage area. In the simulation, the Bloch point reaches its equilibrium position after $\Delta t\approx 0.5\,{\textrm{ns}}$ and does not move for the remainder of $\Delta t_{({\textrm{iii}})}$.

We can repeat steps (i) to (iii) to move the Bloch point past a series of notches, as shown in Fig. 6. Step (iii) is not strictly required to achieve the desired motion past multiple notches. However, it demonstrates several advantages. First, the Bloch point in the free system reaches an equilibrium position in each storage area without an applied current. Hence, successful operation only requires an applied current during a short period of time. This reduces energy consumption of potential devices based on this technology as external energy is only required to change the configuration. Second, the position of the Bloch point at the end of step (ii) is not critical. As long as the Bloch point has moved past the notch, the configuration will automatically converge to a low-energy state with the Bloch point in the desired storage area. This makes the approach more robust and less sensitive to, for example, variations in current pulse duration and notch geometry.

**Figure 7:**
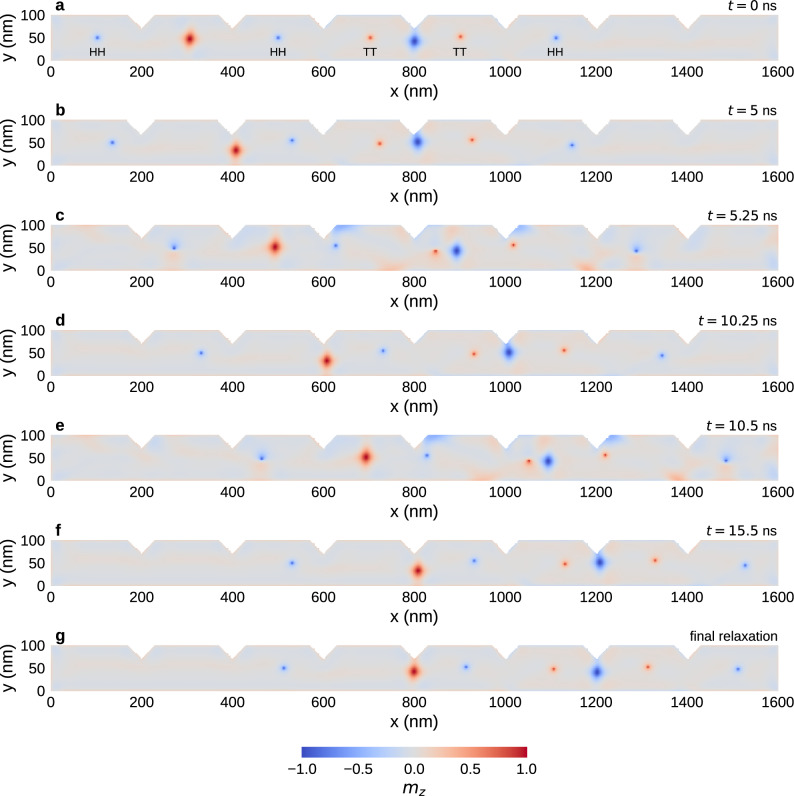
Movement of a configuration containing five Bloch points past two notches by applying a series of current pulses of alternating lower and higher strength. The initial configuration and the distribution of the Bloch points across storage areas is retained.

Figure 7 demonstrates a similar process for a configuration containing multiple Bloch points in the configuration HH-HH-TT-TT-HH (following the notation in Ref.^13^). We simulate a strip with length $l=1600\,{\textrm{nm}}$ containing seven notches. Figure 7a shows the initial configuration with the individual Bloch points labelled.

The two large red and blue blobs at $x\approx 300\,{\textrm{nm}}$ and $x\approx 800\,{\textrm{nm}}$ are antivortices that form between neighbouring Bloch points of the same type and which have significant magnetisation in the *z* direction. A more detailed study of the antivortices and their role in a multi-Bloch-point system is provided in Ref.^13^. For the following discussion, it is sufficient to focus on the Bloch points.

To simplify the simulation process, we only use steps (i) with $\Delta t_{(\textrm{i})}=5\,{\textrm{ns}}$ and (ii) with $\Delta t_{({\textrm{ii}})}=0.25\,{\textrm{ns}}$. We start with (i) a weak current that moves the Bloch points towards the notches where they get trapped to the left of each notch (Fig. 7b). In the simulations it appears that the antivortices experience a repelling force from the Bloch points but apparently do not significantly interact with the notches. Subsequently, we apply (ii) the strong current pulse that pushes each of the Bloch points past the next notch to their right (Fig. 7c). In the configuration at the end of this pulse, at $t=5.25\,{\textrm{ns}}$, we can see that individual Bloch points have travelled different distances during the pulse. After the next weak pulse (Fig. 7d) each of the Bloch points is aligned to the left of the next notch, and all Bloch points have the same spacing to their notch. By applying the weak pulse for a sufficiently long period, we ensure that all Bloch points reach their aligned position near the next notch before applying the next strong pulse. The cycle is repeated once more (Fig. 7e, f), and we can see, at $t=15.5\,{\textrm{ns}}$, that each Bloch point has moved one notch further (in comparison to Fig. 7d) in the direction of the applied current. To demonstrate the stability of the free configuration, we also simulate the free relaxation after the last current pulse. Figure 7g shows the relaxed configuration. We can see that the configuration does not significantly change from the previous step (Fig. 7f), but we observe small shifts of the Bloch points in the $-x$ direction due to the repulsion from the notches and the sample edge.

### T-shape geometry

**Figure 8:**
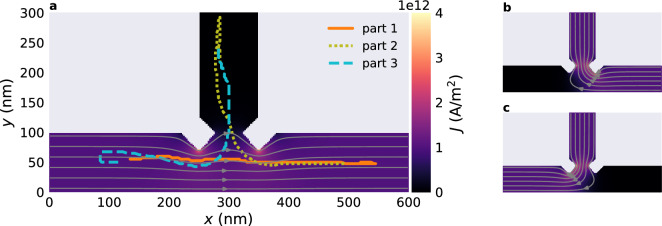
Movement of a single Bloch point through a T-shaped structure when applying a series of current pulses along different directions. The Bloch point is initialised in the left storage area, its position during the simulation is shown with the thick line in (**a**). The Bloch point first moves to the right storage area during part 1 with current applied from the left to the right as shown in the background of (**a**), then to the top during part 2 with current applied from the right to the top as shown in (**b**), and finally back to the left during part 3 with current applied from the top to the left as shown in (**c**). We can see an overshoot of the Bloch point and subsequent relaxation in both part 1 and part 2 of the motion. In part 1 (orange solid line), where the Bloch point is centred in the strip in the *y* direction, the trajectories of forward and backward motion overlap (for $500\,{\textrm{nm}}<x<560\,{\textrm{nm}}$).

Finally, we study a single Bloch point in a T-shaped geometry with three storage areas. The geometry is shown in Fig. 8. It has the three storage areas *left* for $x<200\,{\textrm{nm}}$, *right* for $x>400\,{\textrm{nm}}$, and *top* for $y>150\,{\textrm{nm}}$. They are separated by a total of four notches. We initialise the system in a state where it contains a single Bloch point in the left storage area (at $x\approx 135\,{\textrm{nm}}$). We apply a series of current pulses of varying strength between different pairs of strip ends to move the Bloch point first to the right storage area, then to the top storage area, and finally back to the left storage area.

Each part of the movement consists of three steps: (i) long current pulse with $J_{(\textrm{i})}=0.7\times 10^{12}\,{\text {A/m}}{}^2$ to move the Bloch point to the notches where it gets stuck, (ii) short current pulse with $J_{({\textrm{ii}})}=6.6\times 10^{12}\,{\text {A/m}}{}^2$ to push the Bloch point past the notches, and (iii) free relaxation. The weak pulse (i) is always applied for $\Delta t_{(\textrm{i})}=2\,{\textrm{ns}}$, during which the Bloch point moves towards the notch and gets stuck well before the end of the simulation time. The duration of the strong pulse depends on the part of the motion (see below). For the free relaxation in step (iii) we simulate the time evolution of the system until it reaches an equilibrium state (roughly for $\Delta t_{({\textrm{iii}})}=2{-}4\,{\textrm{ns}}$).

Figure 8a shows the position of the Bloch point during the full cycle. The three parts of the trajectory are shown with a solid orange line for the motion left–right (part 1), a dotted olive line for the motion right–top (part 2), and a dashed cyan line for the motion top–left (part 3). The background in Fig. 8a shows the magnitude of the current density for the current flowing from left to right, streamlines show the current direction. Figures 8b and c show the current flowing from the right to the top and from the top to the left, respectively.

For the motion left–right we apply the strong pulse for $\Delta t_{({\textrm{ii}})}=0.7\,{\textrm{ns}}$. We can see a slight overshoot at the end of the pulse (up to $x\approx 560\,{\textrm{nm}}$). During the free relaxation the Bloch point moves in $-x$ direction to its equilibrium position at $x\approx 500\,{\textrm{nm}}$ near the centre of the right storage area. These two parts of the trajectory overlap in Fig. 8a. For the motion right–top we apply the strong pulse for $\Delta t_{({\textrm{ii}})}=0.5\,{\textrm{ns}}$. Here, we can see a strong overshoot with the Bloch point nearly reaching the top sample boundary at $y=300\,{\textrm{nm}}$. During the free relaxation the Bloch point moves back to the central part of the storage area at $y\approx 200\,{\textrm{nm}}$. For the final motion top–left we apply the strong pulse for $\Delta t_{({\textrm{iii}})}=0.7\,{\textrm{ns}}$. After moving past the two notches, the Bloch point gets deflected in $+y$ direction. This deflection is a result of the uneven thickness of the two layers and the deformed, off-centred Bloch point after it moves past the notches and the junction. We have simulated a system with more similar layer thicknesses and find that the effect decreases. During the free relaxation, the Bloch point moves back to the central part of the storage area at $x\approx 115\,{\textrm{nm}}$. The slightly different initial and final position in the left storage area result from the differences in the initial and final magnetisation configuration due to the series of applied current pulses and the nearly flat energy landscape for a Bloch point inside a storage area. Qualitatively similar behaviour can be seen in Fig. 6, where the free Bloch point also relaxes slightly off-centred.

## Discussion

Our micromagnetic simulations show that Bloch points—which are equilibrium configurations in two-layer FeGe nanostrips—can be moved with spin-polarised currents. In uniform strips, Bloch points move along the current direction without a Hall effect, independent of their type. This is different from behaviour of vortices^18–20^ or skyrmions^21–23^, and also Bloch points in other systems^14^. The straight motion is a special feature of the two-layer system, in which each Bloch point is encapsulated between two vortices with opposite polarisation. The vortices would be subject to deflection in opposite direction but are strongly exchange-coupled across the layer interface. At the Bloch point the forces are compensated, and thus the Bloch point moves in a straight line. Different behaviour has been reported by Gong et al.^14^, who have studied current-induced dynamics of a chiral bobber^24–26^, a skyrmion tube that ends in a Bloch point. They have simulated thin FeGe films with a single material chirality with applied spin-transfer torque and observe a motion of the chiral bobber, and hence also the Bloch point, with a current-dependent Hall effect.

In the system studied here, changes in geometry can be used to modify the motion. We have demonstrated that below a threshold current density a Bloch point can be trapped by a notch. The Bloch point holds its position because the current is pushing it towards the notch constriction, but the Bloch point is repelled from the boundary and does not want to deform, and thus it cannot move past the notch. Using these competing effects, we can choose appropriate current strengths to either neatly align a Bloch point at a notch or move it past a notch. Using a series of current pulses of different strength, we can move an array of Bloch points of different type through strips with multiple notches in a controlled fashion. They retain their initial order and relative distance in terms of empty or occupied storage areas. The additional antivortices between same-type Bloch points do not impede this process, despite not being trapped at the notches themselves. In each storage area we can distinguish between three different local configurations: a HH Bloch point, a TT Bloch point, or no Bloch point. Experimental detection of a Bloch point inside such a storage area is an open question. Electrical detection methods, e.g. based on magneto-resistance effects, could be one possibility.

Furthermore, we have demonstrated that Bloch points can be moved through more complex geometries with multiple possible paths. We have demonstrated this for a T-shaped geometry, where the Bloch point can move along either of the two possible strip ends when being pushed towards the junction depending on the direction of the applied current. In this system, we have added additional constrictions at the junction to create three well-separated storage areas. The Bloch point in the free system reaches an equilibrium position in each of the three storage areas. A weak and subsequent strong pulse can be used to move the Bloch point between storage areas.

In a larger system, these two building blocks—constrictions to restrict the Bloch-point motion and junctions with multiple possible paths—can be combined. Such a system can then host an array of Bloch points and the geometrical constraints can be use to manipulate the array, e.g. re-arrange Bloch points with a series of current pulses between different contacts. Successful operation will likely require more device engineering.

Speculating about potential applications, a simple nanostrip containing a series of Bloch points can be used in a racetrack-like^27^ design, and the two Bloch-point types can be used for binary data representation. The whole array of Bloch points could be moved with spin currents. Retaining equal spacing would not be required because of the two different types that could encode “0” and “1”, which is not the case in skyrmion-based racetrack memories. In a more complex setup we have shown that a series of notches in a strip can be used to create confined “storage areas”. Bloch points can be distributed across these areas, where each area can be in one of three states: occupied with a HH Bloch point, occupied with a TT Bloch point, or not containing a Bloch point—with the potential to realise a ternary storage device. The whole configuration could be moved through this strip using a series of current pulses. Pinning of the Bloch points at the notches helps controlling the movement and ensures that the configuration is retained.

## Methods

### Micromagnetic simulations

We simulate rectangular two-layer nanostrips with opposite chirality (opposite sign of *D*) in the two layers (Fig. 2). We fix the thickness of the two layers to $t_{\textrm{b}} = 20\,{\textrm{nm}}$ for the bottom layer and $t_{\textrm{t}} = 10\,{\textrm{nm}}$ for the top layer. We use a strip width of $w=100\,{\textrm{nm}}$ unless indicated differently. We choose strip lengths that allow for enough space for the Bloch points to move and vary the strip length depending on the number of Bloch points. We use cubic discretisation cells with edge length $2.5\,{\textrm{nm}}$ (for Fig. 3, 6, 7, and 8). For the simulation of a single notch (Fig. 4 and 5) we deviate from the cubic shape and use a slightly smaller cell size in *x* direction ($2.49\,{\textrm{nm}}$) to have an odd number of cells in *x* direction, which improves the staircase approximation of the notch as there is one cell located directly at the notch. The energy equation:

1 $$\begin{aligned} E = \int {\textrm{d}}^3r \left( w_{{\textrm{ex}}} + w_{{\textrm{dmi}}} + w_{\textrm{d}} \right) \end{aligned}$$

contains exchange energy density $w_{{\textrm{ex}}}$, bulk Dzyaloshinskii–Moriya energy density $w_{{\textrm{dmi}}}$, and demagnetisation energy density $w_{\textrm{d}}$. The magnetisation dynamics is simulated using the Landau–Lifshitz–Gilbert equation^28,29^ with currents modeled with the Zhang–Li model^30^:

2 $$\begin{aligned} \frac{\partial {\textbf{m}}}{\partial t} = \gamma {\textbf{m}} \times {\textbf{H}}_{{\textrm{eff}}} + \alpha {\textbf{m}} \times \frac{\partial {\textbf{m}}}{\partial t} -{\textbf{m}} \times [ {\textbf{m}} \times ({\textbf{u}} \cdot \nabla ){\textbf{m}} ] - \beta {\textbf{m}} \times ({\textbf{u}}\cdot \nabla ){\textbf{m}}, \end{aligned}$$

where $\gamma = 2.211 \times 10^5\,{\text {m/As}}$ is the gyromagnetic ratio, $\alpha$ is the Gilbert damping, and

3 $$\begin{aligned} {\textbf{u}}=\frac{P\mu _{\textrm{B}}g}{2eM_{\textrm{S}}(1+\beta ^2)}\ {\textbf{J}} \end{aligned}$$

is the spin-drift velocity. Here, ${\textbf{J}}$ is the electric current density, *P* the polarisation, $\mu _{\textrm{B}}$ the Bohr magneton, *g* the electron g-factor, *e* the electron charge, and $\beta$ the non-adiabatic parameter. Material parameters are based on FeGe^6^: $A = 8.87\,{\textrm{pJ}}\,{\textrm{m}}^{-1}$, $D = 1.58\,{\textrm{mJ}}\,{\textrm{m}}^{-2}$, $M_{\textrm{s}} = 384\,{\textrm{kA}}\,{\textrm{m}}^{-1}$, and $\alpha =0.28$^31^. We use spatially varying current densities ${\textbf{J}}$ of different magnitudes in our simulations and keep the other variables in Eq. (3) fixed to $P=0.5$ and $\beta =2\alpha = 0.56$.

All micromagnetic simulations are performed using Ubermag^32,33^ with OOMMF^34^ as the computational backend and an extension for DMI for crystalclass T^35,36^. We have generalised the Zhang–Li OOMMF extension, in order to simulate current flowing in arbitrary directions. The modified extension is available on GitHub^37^.

As a starting point for all simulations we create rectangular nanostrips containing Bloch points at the desired positions, following a protocol that we developed previously^13^. For rectangular nanostrips we can then directly add the Zhang–Li term with a uniform current density to the dynamics equation and simulate the Bloch point dynamics. For more complex geometries, we first modify the nanostrip to have the desired shape and again minimise the energy to start from a relaxed configuration. Then, we add the Zhang–Li current using the current profile obtained from the finite-elements simulations outlined below and simulate the Bloch point dynamics.

### Locating Bloch points

To analyse the Bloch-point motion we need to locate and track the individual Bloch points. We use a combination of two different methods to precisely locate the Bloch points. Tracking is then simply done based on the distance of Bloch points in two consecutive time steps. In all simulations the Bloch points are clearly separated and their number is kept fixed, hence identifying the individual Bloch points using this simple distance-based method is sufficient.

We first compute the approximate location of the Bloch points (within cell accuracy) based on a classification mechanism that we developed previously^13^. We briefly summarise the method here. Bloch points can be identified as sources and sinks of the emergent magnetic field. To locate Bloch points along one direction we compute the flux of the emergent magnetic field through a series of volumes that we increase along the respective direction. We find quantised jumps in the total flux, whenever the volume includes an additional Bloch point. Based on the sign of the jump we can determine the Bloch-point type. The approximate location of the Bloch point is given by the upper integration boundary. To locate a Bloch point in three dimensions we can repeat this calculation along different directions, where we limit the integration volume along the directions where we have already located the Bloch point. That way we can locate individual Bloch points in a configuration containing multiple Bloch points.

To locate a Bloch point with sub cell size accuracy, we can compute the centre of mass of the emergent magnetic field ${\textbf{B}}^{\textrm{e}}$, defined as:

4 $$\begin{aligned} {\textbf{r}}_{{\textrm{BP}}} = \frac{\int _V {\textrm{d}}^3r\ {\textbf{r}} {\text {div}} {\textbf{B}}^{\textrm{e}}}{\int _V {\textrm{d}}^3r\ {\text {div}} {\textbf{B}}^{\textrm{e}}} \end{aligned}$$

where

5 $$\begin{aligned} B^{\textrm{e}}_i = \epsilon _{ijk} {\textbf{m}} \cdot \left( \frac{\partial {\textbf{m}}}{\partial r_j} \times \frac{\partial {\textbf{m}}}{\partial r_k}\right) . \end{aligned}$$

This method only works if the considered volume contains a single Bloch point. Furthermore, magnetisation tilts at sample boundaries can affect the result. To get reliable results, we first compute the approximate location of all Bloch points. Then, we can compute the precise location of each Bloch point by only considering a small subvolume *V* around the approximate position. In the majority of the work we only use the computationally less expensive locating method with cell-size accuracy.

### Current profile

We use Python libraries, which are part of the FEniCSx project^38–40^, to numerically compute the current profile in non-rectangular nanostrips. Additionally, we use Gmsh^41^ to create the irregular mesh for the geometry.

For an electric conductor, according to the Ohm’s law, the electric current density ${\textbf{J}}$ is defined via:

6 $$\begin{aligned} {\textbf{J}} = \sigma {\textbf{E}}, \end{aligned}$$

where ${\textbf{E}}$ is the electric field and $\sigma$ the electric conductivity. Further, the principle of charge conservation yields:

7 $$\begin{aligned} \nabla \cdot {\textbf{J}} = 0. \end{aligned}$$

According to Maxwell’s equations, in the absence of a time varying magnetic field, the electric field is conservative. Hence, it can be expressed in terms of an electric potential as:

8 $$\begin{aligned} {\textbf{E}} = -\nabla V. \end{aligned}$$

Combining these equations, we obtain:

9 $$\begin{aligned} \nabla \cdot (-\sigma \nabla V) = 0. \end{aligned}$$

Further, we assume as isotropic material, hence the conductivity is a scalar. This gives a Laplace’s equation:

10 $$\begin{aligned} \nabla ^{2}V = 0, \end{aligned}$$

which we can solve numerically after defining suitable boundary conditions.

Figure 2 shows an example for a rectangular nanostrip with a single notch. The calculated current density profile is shown on the top surface. Figure 4c shows a cut plane of the same geometry. We assume a constant current flow through the left and right sample boundary with strength $J_0$ in $+x$ direction and no current flow through any of the other surfaces. Hence, the Neumann boundary conditions can be expressed as:

11 $$\begin{aligned} \frac{\partial V}{\partial n} = {\left\{ \begin{array}{ll} -{J_0}/{\sigma }\qquad &{}\quad \text {if } x=0\,{\textrm{nm}}\\ {J_0}/{\sigma }&{}\quad \text {if } x=600\,{\textrm{nm}}\\ 0&{}\quad \text {else} \end{array}\right. }. \end{aligned}$$

In the example we use $J_0=10^{12}\,{\text {A/m}}{}^2$. The streamlines in Fig. 4c indicate the current direction, colour the magnitude of ${\textbf{J}}$. We obtain a uniform flux in *x* direction in the rectangular parts of the nanostrip. Near the notch, the current profile changes as the current flows around the notch. This leads to a variation in the current density with the maximum at the tip of the notch. To include the current into the finite-difference micromagnetic simulations, we interpolate the simulated current profile onto a cuboidal mesh. Visualisations in Fig. 4 and Fig. 8 are shown on the finite-difference grid used for the micromagnetic simulations.

### Computational science and data analysis

For the preparation of simulation studies and data analysis we make use of the ecosystem of Python-based open-source tools and libraries^42–48^.

## Data Availability

All results obtained in this work can be reproduced from the repository in Ref.^49^ that contains software specifications and Jupyter notebooks^45,50^ to rerun all simulations and recreate all data and plots. In the repository pre-computed datasets are also available.
